# Microstructure-guided design of biopolymer-supported tri-phasic TiO_2_ for sustainable lead and cadmium detoxification

**DOI:** 10.1038/s41598-026-43155-x

**Published:** 2026-03-27

**Authors:** Georgena R. Erian, N. Abdelmonem, Amr Abdelghany, Hoda Abou-Shady, R. O. Abdel Rahman

**Affiliations:** 1 Chemical Engineering Department, Faculty of Engineering, Cairo University, Giza, Egypt; 2 Faculty of Science, Cairo University, Giza, Egypt; 3 Hot Laboratories Center, Egyptian Atomic Energy Authority of Egypt, P. O. No. 13759, Cairo, Egypt

**Keywords:** Polymorph, Eco-friendly nano-composite, Removal performance, Mechanism identification, Chitosan biopolymer, Tri-phasic TiO_2_, Heavy metal; Sustainable water treatment, Chemistry, Environmental sciences, Materials science, Nanoscience and technology

## Abstract

The feasibility of synthesizing tri-phasic TiO_2_ Nano-particles via the sol-gel method and their immobilization within chitosan biopolymer matrix was investigated. Structural characterization using XRD, HR-TEM, FTIR, and UV-vis DRS confirmed the successful formation of a stable hetero-structure consisting of anatase, rutile, and brookite phases (A_34.6_R_56.8_B_8.6_) with strong interfacial interactions within the biopolymer matrix. Reduced direct and indirect band gaps to 2.97 and 2.58 eV, respectively, demonstrated improved optical characteristics under sunlight. The immobilized tri-phasic TiO_2_ Nano-particles within chitosan biopolymer matrix exhibited significantly enhanced sorption performance toward Pb^2+^ and Cd^2+^ ions, reaching maximum removal efficiencies of 99.86% for Pb^2+^ and 97.85% for Cd^2+^ at pH 7, with equilibrium contact times of 90 and 120 min, respectively. According to the Langmuir isotherm model, the maximum removal capacities were 73.67 mg/g for Pb^2+^ and 68.72 mg/g for Cd^2+^. These results highlight the potential of the biopolymer-supported tri-phasic TiO_2_ Nano-composite as a sustainable and effective material for detoxifying heavy metals in water treatment applications.

## Introduction

Clean water is an imperative and vital component of all living organisms, as its chemical composition enables it to act as a universal solvent for numerous nutrients^1^. However, the rapid industrial expansion, modernization, and global population growth have significantly increased water consumption rates and pollution levels^2^. Consequently, water scarcity has emerged as one of the most global challenges of the 21^st^ century^3,4^. It is expected that by 2050, the demand for the water system will increase approximately by an increase of 22–34% as the increasing world population will range between 9.4 and 10.2 billion people^5^. According to the United Nations (UN) World Water Development Report (WWDR) 2023 Edition, more than 57% of the world’s population is expected to experience water scarcity for at least one month each year^6^.

Water pollution is primarily caused by the discharge of untreated industrial effluents into natural water bodies^7^. These effluents often contain barely degradable toxic organic and inorganic pollutants, including heavy metal ions (e.g., Cr, Ni, Cu, Cd, Pb, Zn, Hg, Pt, Co, etc.), pathogenic microorganisms, organic waste (e.g., phenolic compounds, dodecyl benzenesulfonate, etc.), and pigments and dyes^8^. The presence of such pollutants poses severe risks to human as these untreated effluents have the potential to pass through the food chain, posing a significant risk to human health, aquatic ecosystems, and agricultural productivity, as they can accumulate through the food chain and cause long-term environmental damage^7,9^.

To address these challenges, various conventional water treatment techniques have been used for remediation including physical, biological and chemical methods. Physical methods involve screening, filtration, coagulation, flocculation, sedimentation and flotation, while biological treatments rely on aerobic and anaerobic microorganisms to degrade organic matter^9–13^. Chemical treatment methods, such as chemical precipitation, disinfection by using chlorine or ozone or ultraviolet light (UV), sorption and bio-sorption, ultra and Nano-filtration, as well as, reverse osmosis, electrodialysis (ED), ion exchange, and membrane separation, all previous technologies are widely applied for elimination of toxic contaminants from wastewater^9–13^. Among these techniques, sorption is considered one of the most effective and economical approaches due to its simplicity by transferring contaminants from solution onto sorbent surface. In addition to, its high efficiency, ease of operation, easily regeneration of sorbent and producing less harmful byproducts in aqueous solutions^14,15^.

In recent years, several researchers suggest that nanotechnology has emerged as a promising strategy for improving the efficiency of remediating contaminated water. Nano-based materials offer high surface area, high performance, tunable surface chemistry, environmental friendliness and multi-functionality, enabling enhanced sorption and photocatalytic degradation of contaminants^16,17^. Several conventional nanostructured materials, can be used to eliminate the contaminants have dual functions, where their chemical structure allow their use as sorbents and photo-catalysts to eliminate contaminants; these materials including metal oxide Nano-particles (e.g., TiO_2_, ZnO, SnO_2_, etc.), zeolites, activated carbon-based materials, graphene oxides, layered double hydroxides, clay minerals, organic resins, carbon nanotubes (CNTs), and metal organic frameworks (MOFs) and their composites with metal oxides or polymers^18–20^.

Alongside the widespread research interest in Nano-based materials for water remediation, increasing emphasis has been placed on the utilization of natural biopolymers such as cellulose^21,22^, and chitosan^23,24^ owing to their sustainability, abundant availability, biodegradability, biocompatibility, and environmental friendliness^25,26^. Biopolymers provide an environmentally safe foundation for water treatment applications due to the presence of abundant functional groups that enable strong interactions with contaminants. Chitosan is the second most abundant natural biopolymer after cellulose and is unique among natural biopolymers due to the presence of amine groups. It is widely found arthropods, including insects and crustaceans, as well as fungi^25^. The presence of numerous reactive amino groups and hydroxyl groups enables chitosan to play a crucial role in adsorption of heavy metal ions through electrostatic attraction, ion exchange, and chelation mechanisms^27^. Consequently, chitosan has been widely investigated as an efficient adsorbent for the removal of organic pollutants and heavy metals from wastewater^24,28^. Moreover, owing to its polycationic nature, chitosan has shown considerable potential for membrane-based water purification processes, particularly ultrafiltration, which is essential for producing high-purity water^24^. Therefore, the incorporation of biopolymers contributes to enhance the sustainability of polymeric membranes, mitigating membrane waste and extending membrane lifecycles, offering a promising pathway toward environmentally sustainable water purification systems with reduced environmental impact^29,30^. The synergistic benefits of biopolymer–nanoparticle composites in advanced water purification applications have also been highlighted in recent studies. For example, biopolymer-based adsorbents with embedded metal oxide nanoparticles have demonstrated enhanced adsorption performance and rapid removal kinetics toward emerging water pollutants^31^.

As a result, we concentrated on using such an environmentally friendly sorbent to remove toxic metal ions from contaminated water. And because of the increasing interest among the scientists in the value-adding of sourced materials nanotechnology, such as the Nano-composites that are being studied, because of their small size, paramagnetic nature, and low toxicity^32^. The Nano-composites are multi-phasic materials which consist of multiple phases, and at least one of the phases should have dimensions that fall within the range of 10–100 nm and known as the Nano-scale phase^33,34^. The Nano-material can be clay or polymer or metallic and is dispersed in the polymeric matrix to form the polymer Nano-composite material which combines both properties of polymers and Nano-materials (NMs)^35^.

Of the most appealing materials which have intriguing technological characteristics and uses are the Chitosan and titanium dioxide (TiO_2_)^36^. On one hand, the most advantageous characteristics of chitosan is being a natural polymer, biodegradable, non-toxicity, antimicrobial activity, availability, and ease adoptability that is attributed to the presence of reactive amino and hydroxyl groups that facilitate allow for modification to enhance existing properties or add new ones^37^. On the other hand, titanium dioxide (TiO_2_) is widely employed due to its physicochemical, mechanical, and photocatalytic qualities; thermal stability and reactivity; low cost; safe manufacture; and biocompatibility^37,38^.

Numerous studies have reported the use of chitosan/TiO_2_ composites for removal of dyes and organic pollutants from contaminated water. The effectiveness of chitosan/TiO_2_ Nano-composites (CS/TiO_2_) as sorbent for eliminating the cationic dye Methylene Blue (MB) from wastewater is examined by Hari et al.^39^. Karthikeyan et al.^40^ observed that Chitosan/TiO_2_ Nano-composite eliminated the dyes congo red (CR) and rhodamine B (RhB) under visible light. Akash B. et al.^41^ investigated the ability of TiO_2_/chitosan beads to degrade 2, 4-dichlorophenoxyacetic acid and discovered that the degradation efficiency was 92% in batch mode of operation under ultraviolet light. A CS-TiO_2_ membrane with antibacterial and antiproliferative characteristics was developed by Behera et al.^42^. Kaewklin et al.^43^ fabricate a film of CS–TiO_2_ in order to preserve tomatoes. Al-Taweel et al.^44^ reported that the CS/TiO_2_ Nano-composite with a 1:2 ratio (TiO_2_-CS) had the large surface area and shown maximum activity for removing Direct Violet 51 from its aqueous solutions, while Dhanya et al.^45^ found that CS was utilized as an immobilizing agent to enhance the photocatalytic performance of TiO_2_ against methyl orange and alizarin red dye. Significant degradation efficiency against methyl orange dye was also noted by Saravanan et al.^46^. Also, the Cd(II) and Cu(II) ion traces have been eliminated by Mohamed et al.^47^ using a TiO_2_-NPs-bonded CS Nano-layer.

Recent studies have revealed that surface facet engineering, crystallographic phase composition, and interfacial microstructure all have significant impact on the sorption performance of nanostructured materials. It has been demonstrated that rational control over exposed crystal facets and phase junctions greatly increases surface energy, active sorption sites, and metal–surface interactions, which improves the efficiency of heavy metal removal. For instance, facet-dependent metal oxide nanostructures exhibited superior sorption capacities toward Pb^2+^, Cd^2+^, and related metal ions because of improved surface reactivity and optimized coordination environments^48,49^.

Moreover, Modwi et al.^50^ and Khairy et al.^51^ have demonstrated that hetero-structured and multi-phase oxide systems perform better than single-phase materials. This enhanced performance can be attributed to several factors, including improved charge separation, accelerated electron transport across phase boundaries, enhanced reaction kinetics, and synergistic interfacial effects. Further evidence provided by Zango et al.^52^, Algreiby et al.^53^, and Alqarni et al.^54^ indicates that microstructure-guided design is essential for regulating the binding affinity of heavy metals, as well as ensuring sorption stability and optimizing reaction kinetics. This evidence is derived from molecular-level structural studies. Collectively, these findings emphasize the crucial rule of surface facet engineering and polymorphic phase integration in developing high-performance sorbents for sustainable water treatment applications.

Despite these advances, most of the reported studies rely on single-phase or bi-phasic TiO_2_ systems and primarily target organic contaminants. On the other hand, the current study presents a microstructure-guided design for effective heavy metal detoxification that is based on immobilization of tri-phasic TiO_2_ (anatase, rutile, and brookite) hetero-structure within a chitosan biopolymer matrix. The coexistence of three TiO_2_ polymorphs with well-defined phase ratios is expected to promote interfacial charge transfer and improve sorption and photocatalytic performance under sunlight. The current study also aimed to develop a multifunctional Nano-composite material to meet the needs of high removal capacity, cost-effectiveness, nontoxicity and ecofriendly sustainable Nano-composite material with a small footprint. For this purpose, a novel biopolymer-supported tri-phasic TiO_2_ nanocomposite is synthesized via a sol–gel approach, forming a stable hetero-structure with enhanced interfacial interactions. The focus is to investigate the feasibility of eliminating heavy metals such as Pb^2+^ and Cd^2+^ ions from contaminated water under sunlight by using the ecofriendly tri-phasic polyform TiO_2_ immobilized in the chitosan biopolymer, which represents the key novelty of this study and highlights its potential for sustainable water treatment applications.

In this respect, the structural, morphological, and optical properties of this Nano-composite were identified by the collecting X-ray diffraction (XRD), high-resolution transmission electron microscopy (HR-TEM), Fourier transform infrared (FTIR) spectroscopy, UV–Visible Diffusion Reflectance Spectroscopy (UV–Vis DRS Spectrophotometer). The study investigates the effect of numerous variables, including pH, contact time, and initial concentration on the removal capacity and efficiency of the material. Finally, the results were mathematically analyzed to identify the elimination reaction kinetics and mechanisms.

## Materials and methods

### Chemicals

The main raw materials which used in synthesis of Chitosan/TiO_2_ Nano-composite are Chitosan as a biopolymer (deacetylation 75%; and low molecular weight of 50,000–190,000 Da), Titanium (IV) Isopropoxide (TTIP) as the precursor of TiO_2_ (27.5–28.5%TiO_2_) was acquired from Alpha Chemika, India. Absolute Ethanol 99.5% was used as solving solution for TiO_2_ synthesis and was obtained from Sigma Aldrich, Germany and Nitric Acid (HNO_3_) 69.5% was from Carlo Erba Reagents Group, France. Glacial Acetic Acid AR was used as solving solution for chitosan synthesis and was obtained from Alpha Chemika, India. Deionized water was used throughout this work. Stock simulated contaminated solutions were prepared by dissolving the appropriate amount of analytical grade ion salts in distilled water, the ions salts as lead and cadmium chloride were purchased from Koch-light Laboratories. The initial pH of the solutions was adjusted to a given value by using a solution of 0.1 M NaOH and 0.1 M HCl were obtained from Alpha Chemika, India.

### Methods

#### TiO_2_ nano-particles synthesis

The TiO_2_ nano-particles was synthesized using Sol-gel (SG) Process as described in^55^, which involves several steps and requires mixing of two different solutions. These solutions are known as precursor and hydrolysis solutions^41^. In our procedure, the precursor solution is a mixture of 10 mL of Titanium (IV) Isopropoxide (TTIP) and 30 mL of Ethanol Absolute Grade, the mixture was stirred for 15 min by magnetic stirrer at temperature 30–40 °C. The hydrolysis solution prepared by stirring a mixture of 150 mL deionized water and 3 mL of Nitric Acid HNO_3_ in order to control the sol gel reactions (hydrolysis and condensation). The hydrolysis solution was added dropwise into the precursor solution under constant stirring for around 4 h at temperature 60–70 °C. In this step the high viscous solution was obtained then converted to form Yellowish cleared transparent solution. Let the previous solution (the formed gel) cooled in room temperature while continues stirring it for 30 min. The formed gel kept in dryer for drying at 110 °C for 7 h to form white precipitation. The calcinations for the obtained white precipitation was in oven at 550 °C for 4 h to obtain the TiO_2_ nano-particles.

#### Biopolymer chitosan preparation

Let 1% W/V of low molecular weight Chitosan with 75% deacetylation was dissolved in 1% V/V glacial acetic acid solution with continuous stirring for 1 h at room temperature as described in^56^.

#### Immobilization of TiO_2_ nano-particles in biopolymer matrix

From the prepared Chitosan solution, 1 gm of prepared TiO_2_ nano-particles magnetically Stirred with 25 mL of Chitosan solution for 1 h. Then the solution was dried at temperature 70 ℃ for 4 h to obtain a composite. The formed composite was rinsed with NaOH solution (0.1 M) and then washed twice with distilled water. After that the obtained composite was dried again at 70 ℃ and then grounded^57^.

#### Material characterizations

High-Resolution Transmission Electron Microscopy (HR-TEM) was used to investigate the morphological studies and the images were obtained using HR-TEM (JEOL, JEM-2100, Tokyo, Japan) with an accelerating voltage of 200 KV. The Crystallinity, phase and structure analysis were characterized by X-Ray Diffraction Analysis (XRD Analysis) were investigated using XRD analyzer (Bruker D8 advance X-ray diffractometer, Germany). The diffracted pattern was collected using: Cu-kα radiation (λ = 1.54 Å) with a scanning rate of 0.05°/s and a range of (2θ) from 4.00° to 79.91°. Fourier Transform Infrared Spectroscopy (FTIR) was employed for spectral analysis and functional groups identification. FTIR characterizations were obtained by using a Fourier Transform Infrared Spectrometer (Burker VERTEX 80, Germany) combined Platinum Diamond ATR, comprises a diamond disk as that of an internal reflector in the range of 400–4000 cm^− 1^ with resolution 4 cm^− 1^ and refractive index 2.4. Optical properties, especially the optical band gap (E_g_) of Nano photo-catalysts were obtained using UV-Visible Diffusion Reflectance Spectroscopy (UV–Vis DRS). The investigation was performed by diffuse reflectance spectroscopy (JASCO V-570 UV–Vis Spectrophotometer) in wavelength range of 190 nm to 2500 nm at room temperature.

### Determination the optical band gap

Optical properties, especially the optical band gap (E_g_) of Nano-photo-catalysts were obtained using UV-Visible Diffusion Reflectance Spectra which is usually determined by UV–Vis DRS Spectrophotometer.

The Kubelka–Munk function as illustrated in Eq. (1) is well-known for its effective method of converting DRS spectra to a comparable absorption coefficient, which is commonly used to identify the band gap of powder samples^58^.

1 $$f\left(R\right)=\frac{K}{S}=\frac{{(1-R)}^{2}}{2R}=\alpha$$

where, $f\left(R\right)$ is called Kubelka–Munk function, R is relative diffuse reflectance at each wavelength of thick sample referred to non-absorbing standard^58^, while K and S are the absorption and scattering coefficients, respectively and α is the equivalent absorption coefficient.

Tauc’s equation proposed a relationship between the equivalent absorption coefficient and the energy of the photons as expressed by the following Eq. (2)^58,59^:

2 $${(\alpha.hv)}^{\frac{1}{n}}=A(hv-{E}_{g})$$

where, h is Planck’s constant, v is frequency, hv is incident photon energy, E_g_ is a band gap energy of the semiconductor, A is proportional constant, and n represents the type of electron charge transition which causing the optical absorption and its value can be equal to $\frac{1}{2},\frac{2}{3},2and\frac{1}{3}$ for indirect allowed transition band gap, direct forbidden, direct allowed and indirect forbidden transitions, respectively. In case, the TiO_2_ rutile (R) and brookite (B) phases are known as direct band gap semiconductor, whereas anatase (A) phase is indirect band gap semiconductor^60^. And by putting the F(R) instead of (α) in Tauc’s equation that subsequently forms the following Eq. (3). The band gap was determined by interfering tangential lines with the abscissa and displaying a plot of ${\left[f\left(R\right)hv\right)]}^{\frac{1}{n}}$versus photon energy ($hv$)^59^.

3 $${\left(f\left(R\right)hv\right))}^{\frac{1}{n}}=A(hv-Eg)$$

### The performance measures

All experiments were conducted to study the effect of different operating conditions on the removal process. Batch technique was employed to determine the optimum removal pH conditions, contact time and initial concentration. The removal of inorganic contaminates, i.e. Pb^2+^, Cd^2+^ ions onto the immobilized TiO_2_ in biopolymer were carried out by adding Chitosan/TiO_2_ Nano-composite at a fixed dosage of 3.33 g/L into a beaker containing 30 mL of 100 mg/L Pb^2+^ ions at 25 ℃ and the same steps for other heavy metal contaminate Cd^2+^. The investigated variables including of pH (3–11), contact time (5–120 min) and initial concentration (50–250 mg/L) in a batch system. The concentrations of Lead ions (Pb^2+^) and Cadmium ions (Cd^2+^) in the solutions after the contact with hetero-structure Nano-composite were measured by using an Inductively Coupled Plasma–Optical Emission Spectrometer (ICP-OES) of the model Agilent 5100 Synchronous Vertical Dual View (SVDV).

The transient removed amount of the metal ions (q_t_; mg/g) was investigated and calculated as illustrated in Eq. (4):

4 $${q}_{t}=({\mathrm{C}}_{\mathrm{o}}-{C}_{t})\frac{\mathrm{V}}{\mathrm{m}}$$

where, V is the volume of contaminated solution (L), m is the weight of hetero-structure Nano-composite (g) and C_o_ (mg/L) and C_t_ (mg/L) are the initial and transient concentrations of contaminant in the solution before and after removal, respectively. When the contact time t is equal to or greater than the equilibrium time, the removal capacity at equilibrium (q_e_; mg/g) can be calculated by using Eq. (5) and the removal efficiency percentage E% of the contaminant was investigated and calculated as illustrated in Eq. (6):

5 $${q}_{e}=\left({C}_{o}-{C}_{e}\right)\frac{v}{m}$$

6 $$\mathrm{E}\mathrm{\%}=\left(\frac{{C}_{o}-{C}_{e}}{{C}_{o}}\right)\mathrm{*}100\mathrm{\%}$$

where, C_e_ is the equilibrium concentration of the contaminant in the solution (mg/L). Therefore, the removal capacity and the removal efficiency will be used as performance measures to evaluate the suitability of the prepared hetero-structure Nano-composite material in removing Lead and Cadmium ions from their aqueous solutions.

#### Effect of pH

The effect of initial pH of the solution on the removal of Pb^2+^ and Cd^2+^ heavy metal contaminants onto the immobilized TiO_2_ in chitosan biopolymer material were investigated by varying the pH of solutions in range from 3 to 11. The batch experiments were carried out by adding the hetero-structure Chitosan/TiO_2_ Nano-composite at a fixed dosage of 3.33 g/L for volume 30 mL of solution with initial concentration of 100 mg/L for Pb^2+^ and Cd^2+^ ions contaminated solutions then shacked for 2 h contact time at 150 rpm at room temperature. It is evident that the amount of removal strongly depends on pH solution of media in order to evaluate the optimum pH of removal process. The distribution coefficient (K_d_, L/g) of Pb^2+^ and Cd^2+^ ions between the solid and liquid phases was calculated by the following Eq. (7)^61,62^:

7 $${K}_{d}=\left(\frac{{C}_{o}-{C}_{e}}{{C}_{e}}\right)\frac{v}{m}$$

To identify the mechanism of the removal process, the number of H^+^ emitted from the surface of Nano-composite material must be determined. The removal data are regressed linearly to the well-known Kurbatov equation. Consequently, the following Eq. (8) is used for determining the stoichiometry of the exchange reaction^63^:

8 $$Log{\rm{ }}{K_d} = {\rm{ }}\log {\rm{ }}{K_{ex}}{\rm{ }} + {\rm{ }}n{\rm{ }}p{H_{eq}}$$

where, K_ex_ is the exchange constant and n is the slope of straight line^64^ which denotes the (H^+^ /M^z+^) stoichiometry of the exchange reaction.

#### Effect of contact time

Important considerations for developing wastewater treatment technology are the rate at which contaminants are removed and the specification of the equilibrium time^65^. The influence of contact time on removal behavior of Pb^2+^ and Cd^2+^ by using the immobilized TiO_2_ in biopolymer was studied at different contact time varying from 5 min to 3 h, initial concentration 100 mg/L, solution pH of 7, and temperature of 25 °C with specific Chitosan/TiO_2_ Nano-composite material dosage.

#### Effect of initial concentration

The influence of an initial concentration on removal behavior for Pb^2+^ and Cd^2+^ by using the immobilized TiO_2_ in biopolymer was determined by preparing different initial concentrations of contaminants solution ranging from 50 to 300 mg/L at the same pH, time and sorbent dosage.

### Study of sorption isotherms

Sorption isotherms are mathematical models that describe the distribution of the contaminant species among liquid and sorbent, based on a set of assumptions that are mainly related to the heterogeneity/homogeneity of sorbents^66^. Sorption isotherms used for analyzing of removal process data according to description of the design for sorption system through how the contaminant interacts with sorbents, how equilibrium is established between sorbed contaminants on the specific sorbent for determination the maximum removal capacity of sorbent and its efficiency^67^ and the residual contaminants in the solution during the surface removal process. The type of sorption can be determined using a variety of isotherm models. The Equilibrium sorption isotherm models including Langmuir, Freundlich and Temkin models^68^ were used to elucidate the removal capacity of contaminants onto the sorbent Nano-composite, and provide insights into the mechanism of sorption.

#### Langmuir model

The Langmuir isotherm assumes of that monolayer sorption onto a surface containing a finite number of sorption sites of uniform strategies with no transmigration of sorbate in the plane surface and a homogenous surface energy distributed is considered^69^. The mathematical equation of this model can be described by of the following Eq. (9)^70^:

9 $${q}_{e}=\frac{{q}_{max}\cdot{k}_{L}\cdot{c}_{e}}{1+{k}_{L}.{C}_{e}}$$

where, q_e_ (mg/g) and C_e_ (mg/L) represent the observed removal capacity of sorbent and equilibrium concentration of contaminants in solution at equilibrium time, respectively; while q_max_ (mg/g) and k_L_ (L/mg) are the maximum removal capacity of sorbents and the equilibrium constant of the Langmuir model related to the affinity of the binding sites, respectively. Dimensionless sorption intensity (R_L_), which may be computed using the following formula in Eq. (10), can be used to assess the favorability of the Langmuir isotherm^71^.

10 $${R}_{L}=\frac{1}{1+{k}_{L}.{C}_{m}}$$

where, C_m_ is the maximum initial concentration of contaminant solution and R_L_ is terms as the separation factor and represents the shape of the isotherm, which can be either unfavorable (R_L_ > 1), linear (R_L_ = 1), favorable (0 < R_L_ < 1), or irreversible (R_L_ = 0) depending on R_L_.

#### Freundlich model

The Freundlich isotherm model is an empirical equation that is very useful and accurate which can be used to identify the non-ideal sorption based on the assumption of that the removal process takes place on heterogeneous surfaces^71^, in which the energy term in the Langmuir equation varies as a function of the surface coverage. Freundlich isotherm is more precise because of consideration the exponential distribution of active sites and their energies for low sorbed species occupancy. It is superior to the Langmuir isotherm in that it incorporates physical species sorption. The Freundlich isotherm can be expressed as below Eq. (11)^72^:

11 $${q}_{e}={k}_{f}.{{C}_{e}}^{\left(\frac{1}{n}\right)}$$

where, k_F_ (L/g) is the Freundlich constant related to removal capacity and n is the empirical constant related to sorption intensity of the sorbent. The magnitude of 1/n signifies the favorability of the removal process. The 1/n values can define the order of isotherm. i.e., irreversible (1/*n* = 0), favorable (0 < 1/*n* < 1), unfavorable (1/*n* > 1) and 1/*n* = 1 indicates a linear adsorption.

#### Temkin model

A portion of Temkin isotherm specifically takes into consideration the sorbent and sorbate indirect interactions and their effect on sorption process. The model assumes that the function of the temperature of every molecule in the layer will drop linearly with coverage rather than logarithmically, neglecting the extremely low and high concentration values^73^. The equation suggests that, up to a certain maximal binding energy, the binding energies in the derivation are uniformly distributed. The model can be described by the following Eqs. (12) and (13)^74^:

12 $${q}_{e}={B}_{T}.\mathrm{ln}({A}_{T}.{C}_{e})$$

13 $${B}_{T}=\frac{R.T}{b}$$

where, B_T_ is the Temkin Constant, R is universal gas constant (8.314 J/ mol. K), T (K) is the absolute temperature at 298 K, b (J/mol) is the heat of sorption and A_T_ (L/g) is the Temkin equilibrium binding constant.

### Kinetic study

The kinetic mechanism for the removal process of Pb^2+^ and Cd^2+^ by the immobilized TiO_2_ onto chitosan biopolymer matrix has been studied using four kinetic models: the pseudo-first order, pseudo-second order, Elovich Model, and Intra-particular diffusion Model. Kinetic studies are beneficial to assess the sorbent quality and distribute information regarding the potential removal mechanism, which includes mass transfer, chemical reactions and diffusion (external, intraparticle, and bulk)^75^. The non-linear regression correlation coefficient R^2^ was used to determine the best fit model, which measures how closely the predicted values from a prediction model match experimental data. An elevated R^2^ value signifies that the model well described the kinetics removal mechanism of Pb^2+^ and Cd^2+^.

#### Pseudo-first order

The pseudo-first order model (PFO) is defined as Lagergren rate equation. The PFO model postulates that the diffusion of sorbed molecules on the sorbent surface determines the rate of removal at which depends on the nature of the sorbates and the available sites in the sorbent for physi-removal process^76^. The pseudo-first order kinetic model assumes that the rate of change of the solute uptake with time is directly proportional to the difference in the saturation concentration and the amount of sorption with the time, i.e. the rate of occupation of sorption sites is directly proportional to the number of unoccupied sites^77^. If the sorption satisfies the pseudo-first-order model, it indicates that the sorption of the contaminant is primarily physical^68,76^. The non-liner form of PFO model can be expressed as Eq. (14)^78^:

14 $${q}_{t}={q}_{e}(1-{e}^{-{K}_{1}t})$$

where, q_e_ (mg/g) and q_t_ (mg/g) are the removal capacity at equilibrium time and theoretical removal capacity at time t (min), respectively and k_1_ (min^− 1^) is the rate constant coefficient of sorption for Pseudo- First Order Kinetic model (min^− 1^).

#### Pseudo-second order

The Ho and McKayrate equation refer to reaction equation for pseudo-second order (PSO). In PSO model postulates that sorption sites occupation rate is proportional to number of empty sites squared. Also, it assumes that the rate limiting step may be chemi-removal process involving valence forces through sharing or exchange of electrons between sorbate and sorbent^68^. If the sorption satisfies the PSO model, it indicates that the sorption of the contaminant is mainly controlled by chemical reactions with the aid of ion exchange, coordination reactions, hydrogen bonding, π-π interactions^68^. The non-linear form of the PSO kinetic model was expressed as Eq. (15) and (16)^79^:

15 $${q}_{t}=\frac{h.t}{1+{k}_{2}{q}_{e}t}$$

16 $$\mathrm{h}={{\mathrm{k}}_{2}\mathrm{q}}_{\mathrm{e}}^{2}$$

where, h (mg/g. min) is the initial removal rate and k_2_ (g/mg. min) is the rate constant of PSO model.

#### Elovich model

The Elovich Model is frequently used to simulate how gases, metal ions, and organic contaminants chemisorb in various sorbents and this model considered as empirical and lacks clear physical definitions. Several studies claim that Elovich’s model is appropriate for evaluating chemisorption mechanism on high heterogenetic sorbent surfaces. It is based on the kinetic principle assuming that sorption sites increase exponentially with the progress of the sorption, implying multilayer sorption, and that each layer presents different activation energy for chemisorption^80^. The model assumes that the sorption kinetics at low surface coverage are not significantly impacted by desorption or interactions amongst the sorbed species, and that the actual solid surfaces are energetically heterogeneous. Therefore, a rise in the number of sorbed species is exponentially correlated with a decrease in the rate of sorption. This model describes chemisorption kinetics. The Elovich equation expressed as following Eq. (17)^81^:

17 $${q}_{t}=\frac{1}{\beta}\mathrm{ln}(\alpha\beta t+1)$$

where, β (g/mg) and α (mg/g. min) are desorption constant and the initial sorption rate, respectively.

#### Intra-particle diffusion model

Although the removal process mechanism may be effectively determined using the PFO and PSO kinetic models, the diffusion mechanism of sorbates molecules on sorbents is not clarified by these models. The elimination process is best studied using the intraparticle diffusion model. The intraparticle diffusion model proposed by Weber and Morris^82^ Eq. (18):

18 $${q}_{t}={k}_{i}{t}^{\frac{1}{2}}+C$$

where, k_i_ (mg/g min^− 0.5^) is the intraparticle diffusion rate constant, t^1/2^ is the half-life and C (mg/g) is constant related to the boundary layer thickness. When C is passing through the origin (C = 0) means that the intraparticle diffusion model is a rate-controlling step which control the removal rate. On the other hand, without passing through the origin (C > 0), it indicates that the sorption is influenced by other processes which means that the external mass transfer and intraparticle diffusion are considered the rate-controlling steps for sorption^68^.

The removal process is exclusively controlled by intraparticle diffusion; otherwise, if it is controlled by interfacial and intraparticle diffusion, the slope of these linear segments in each phase is used to determine the rate constant k_i_. Higher values of a rate constant (k_i__1_) in the initial phase comparing to rate constant (k_i__2_) suggest that the external binding sites of sorbent are rapidly occupied during sorption. Therefore, the contaminants molecules tend to enter interior pores of sorbent which in this stage the sorption shifts into second phase of sorption^83^.

## Results and discussion

### Characterization

#### XRD analysis

**Fig. 1:**
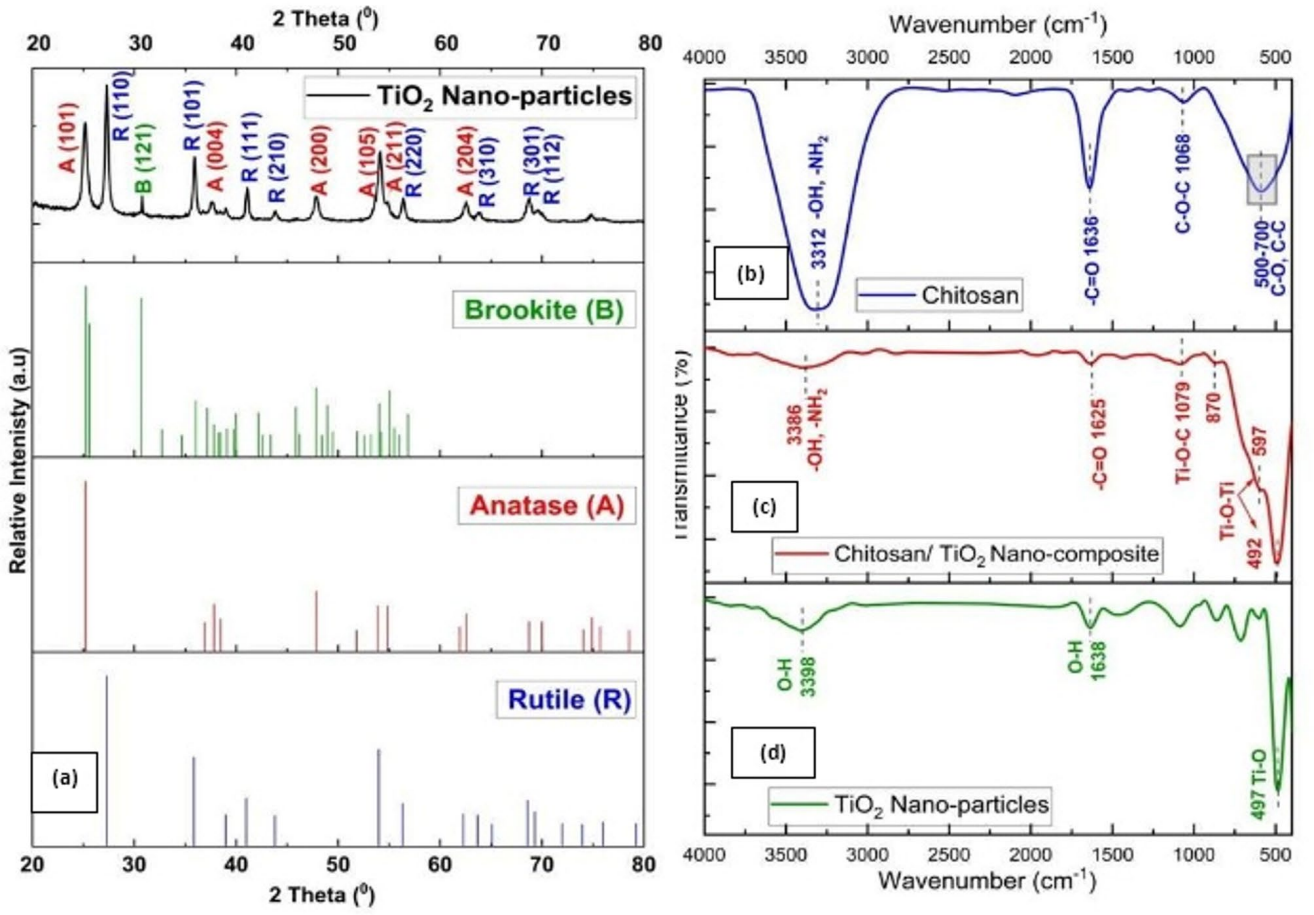
Mineralogical and chemical composition characterizations: (**a**) TiO_2_ nanoparticle XRD pattern; (**b**) chitosan FTIR spectra; (**c**) chitosan/TiO_2_ nanocomposite FTIR spectra; and (**d**) TiO_2_ nanoparticles FTIR spectra.

The XRD pattern illustrated in (Fig. 1a) shows that the prepared TiO_2_ is tri-phasic, where anatase (A), rutile (R) and brookite (B) are detected. The diffraction peaks at 25.224, 37.459, 47.848, 53.867, 54.848 and 62.542° are indexed to the (101), (004), (200), (105), (211) and (204) diffraction planes of anatase (A) phase^60^. The Nano-crystalline structure of the TiO_2_ Nano-particles anatase (A) phase shows a tetragonal unit cell with dimensions a = 3.799, b = 3.799, and c = 9.509 Å.

The diffraction peaks located at 27.289, 35.843, 41.156, 43.953, 53.982, 56.438, 62.283, 63.671, 68.565 and 69.279° are indexed to the (110), (101), (111), (210), (211), (220), (002), (310), (301) and (112) diffraction planes of tetragonal rutile (R) phase^60,84^. These XRD patterns were consistent with the standard XRD data of the anatase (A) and rutile (R) for TiO_2_ phases according to JCPDS No. 021-1272 and JCPDS No. 021-1276, respectively. In addition, the existence of brookite (B) was indicated by the diffraction peak corresponding to the (121) diffraction planes^84^ of orthorhombic brookite (B) phase which appears at 30.679° in XRD pattern according to JCPDS No. 029-1360, and is not overlapping with any other peak from anatase (A) or rutile (R)^85,86^. According to JCPD No. 029-1360, the detection of brookite by the existence of its twin peak at 25.34° (120) and 25.7° (111) was extremely difficult because the anatase main diffraction peak (101) at 25.224° overlapped with the brookite peaks (120) and (111), which were at 25.34° and 25.7°, respectively^85^. The ratio of the identified phases are as follow A:R:B = 0.345:0.568:0.086, hence the polymorph TiO_2_ is expressed as A_34.6_R_56.8_B_8.6_. The coexistence of anatase, rutile, and brookite phases is expected to generate multiple heterojunction interfaces, which play a crucial role in enhancing surface reactivity and sorption performance toward heavy metal ions.

#### Fourier-transform infrared spectroscopy (FT-IR) (spectral analysis)

The FT-IR spectra of tri-phasic TiO_2_ Nano-particles, hetero-structure Chitosan/TiO_2_ Nano-composites, and biopolymer Chitosan are presented in (Fig. 1b–d). The spectrum of tri-phasic TiO_2_ Nano-particles exhibits a characteristic band of TiO_2_ at 497 cm^− 1^, corresponding to the stretching vibration of (Ti–O) bond. The broad absorption bands observed at 3398 cm^− 1^ and 1638 cm^− 1^ are attributed to the stretching and bending vibrations of hydroxyl groups (–OH) associated with surface-adsorbed water molecules on TiO_2_ Nano-particles surface as commonly previous reported (Fig. 1.d)^45,87^.

The FT-IR spectrum of chitosan (Fig. 1b) exhibited a broad band at 3312 cm^− 1^ that could be attributed to the overlapping stretching vibrations of hydroxyl (–OH) and amino (–NH_2_) groups. The characteristic amide I band observed at 1636 cm^− 1^ is associated with (–C = O) stretching vibration coupled with (–N–H) bending. Furthermore, the band at 1068 cm^− 1^ is attributed to (–C–O–C) stretching vibrations of the polysaccharide structure backbone in the glucose unit of chitosan^38,88^. Additional bands observed in the range of 600–700 cm^− 1^ are considered as the fingerprint bands arising from skeletal vibrations of the chitosan molecules, including the stretching modes of C–C and C–O out of plane bending modes associated with the pyranose sugar rings, confirming the saccharide structure of biopolymer chitosan^89,90^.

The hetero-structure chitosan/TiO_2_ Nano-composite was illustrated in (Fig. 1.c) exhibits characteristic absorption bands of both biopolymer chitosan and tri-phasic TiO_2_ Nano-particles, indicating the successful incorporation of TiO_2_ nanoparticles within the biopolymer matrix. A noticeable 11 cm^− 1^ shift of the amid I band from 1636 cm^− 1^ pristine chitosan to 1625 cm^− 1^ in the Nano-composite was observed, suggesting strong interactions between the carbonyl (–C = O) and amino (–NH_2_) groups of chitosan and the TiO_2_ Nano-particles surface as reported previous^38^. Additionally, the broad (–OH)/(–NH_2_) stretching band shifts from 3312 cm^− 1^ to 3386 cm^− 1^ with deceasing in intensity, indicating the involvement of these functional groups in interfacial interactions, likely through hydrogen bonding or via electrostatic interaction of (N–H–O–Ti) linkages at peak 3386 cm^− 1^^46^. The identification of chitosan within the chitosan/TiO_2_ Nano-composite is not solely based on –OH stretching region, which may overlap with hydroxyl groups originating from surface-adsorbed water on TiO_2_. Instead, the characteristic backbone structure of chitosan is clearly evidenced by distinct carbon- and nitrogen-related vibrational modes. These include the amide I band at 1625 cm^−1^, the C–O–C stretching vibration at ~ 1079 cm^−1^ corresponding to the polysaccharide backbone, and the appearance of peak at 870 cm^− 1^ was attributed to (C–H) out of plane bending and ß-glycosidic ring vibrations, which are signs of maintained chitosan structure^91^. All of these features are absent in pristine TiO_2_ and therefore provide reliable evidence for presence and structural integrity of chitosan in the Nano-composite.

Furthermore, the band that corresponds to the (Ti–O–C) bond emerged at 1079 cm^− 1^, suggesting chemical interaction between chitosan biopolymer and TiO_2_ Nano-particles rather than merely absorbed into biopolymer matrix. Also, the incorporation of TiO_2_ Nano-particles in chitosan matrix leads the appearance of peaks around 492 and 597 cm^− 1^ are assigned to the (Ti–O–Ti) bending and stretching vibration of (Ti–O–Ti) bonds, respectively^92^, and in another hand the peak at 597 cm^− 1^ indicates the immobilization of Titanium in Chitosan biopolymer matrix^93^. While, the peak observed at 492 cm^− 1^ indicates an interaction between Ti Lewis Site and amino groups (–NH_2_) which exist in chitosan biopolymer matrix^38^. These findings collectively support the formation of a chemically interacted chitosan/TiO_2_ Nano-composite and suggest effective immobilization of TiO_2_ Nano-particles within chitosan matrix. Therefore, these strong interfacial interactions are expected to facilitate cooperative binding of Pb^2+^ and Cd^2+^ ions through surface complexation and chelation mechanisms.

#### Transmission Electron Microscopy (TEM) (morphological studies)

**Fig. 2:**
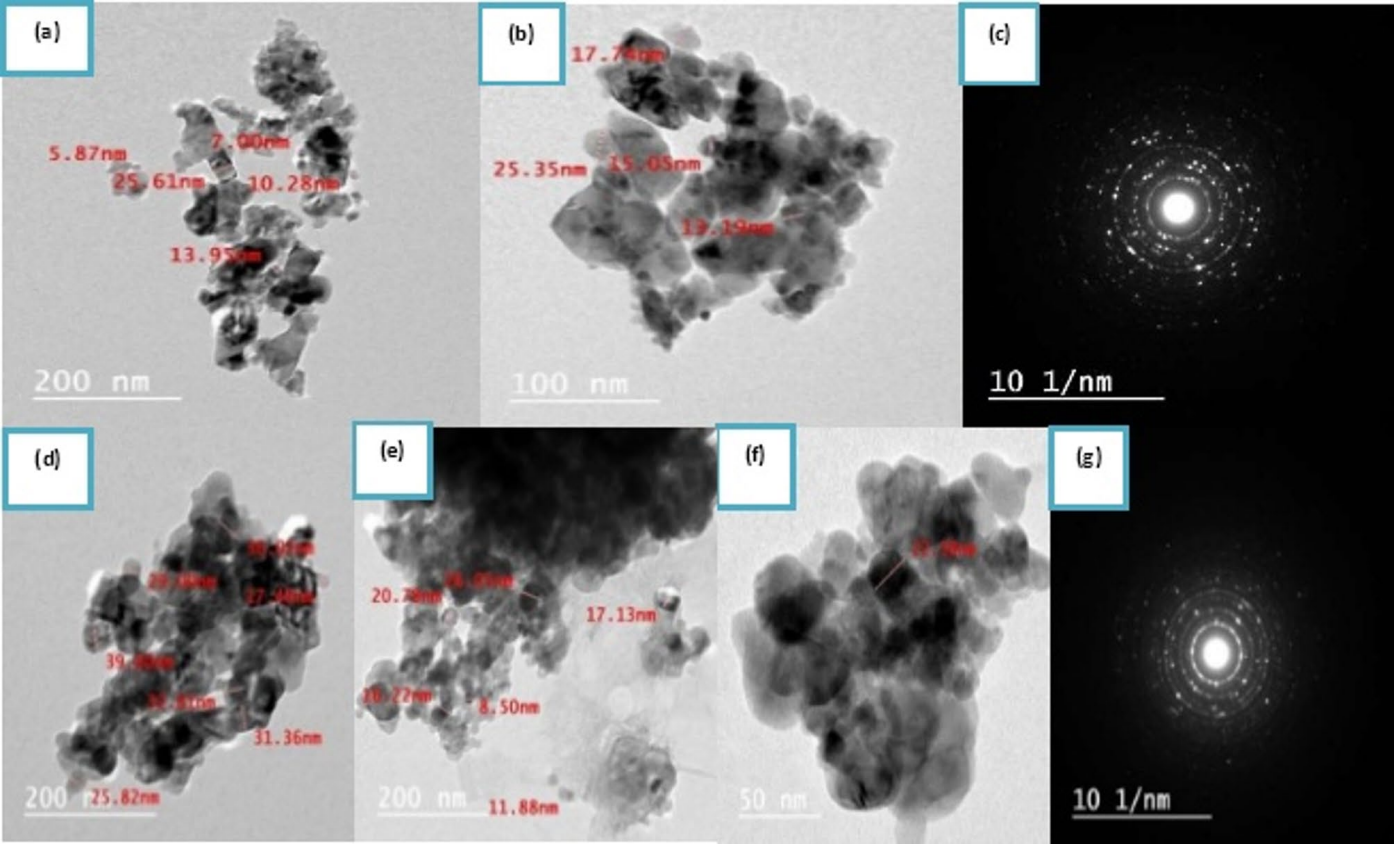
Nano-morphology and crystallographic characteristics of the TiO_2_ Nano-particles: (**a**) low magnification TEM image; (**b**) high magnification TEM image; (**c**) lattice interlayer analysis and selected area electron diffraction (SAED) pattern, as well as the Nano-morphology and crystallographic characteristics of the chitosan/TiO_2_ Nano-composite: (**d**,**e**) low magnification TEM image; (**f**) high magnification TEM image; and (**g**) lattice interlayer analysis and SAED pattern.

The Transmission electron microscopy (TEM) was employed to investigate the morphology, particle size distribution, and crystallographic characteristics of tri-phasic TiO_2_ Nano-particles and chitosan/TiO_2_ Nano-composites as indicated in (Fig. 2). Titanium dioxide particles have irregular shaped particles, where particle agglomeration is evident with a particle size in the range 25.6–7 nm as observed in (Fig. 2a). The magnification of the Nano-graph shown in (Fig. 2b) reveals that the prepared material composed of Nano tetragonal and orthorhombic particles are agglomerated with identified facets in certain orientations, which can be attributed to the exposure high-energy and hydrophilic nature of the Nano-particles during the drying process^94^. The corresponding optical diffraction pattern for TiO_2_ Nano-particles obtained by the selected area electron diffraction (SAED) is illustrated in (Fig. 2c). The tri-phasic polymorph nature of the prepared material is evident, where concentric rings are clearly displayed^95^. The rings are formed of separated dots, which can be related to the presence of the agglomerated TiO_2_ particles^95^. The lattice fringes have interlayer distances in the selected area equal to 3.3 and 8.1 Å. The first is attributed to the (1 1 0) plane in the Rutile phase lattice, and the latter is assigned to the brookite^96^.

However, as shown in (Fig. 2d,e), the chitosan/TiO_2_ Nano-composite exhibits particle agglomeration with a particle size in the range of 39.6–8.5 nm and also shows that TiO_2_ Nano-particles are immobilized in the chitosan matrix, indicating the successful preparation of Chitosan/TiO_2_ Nano-composite. The magnification of the Nano-graph for Chitosan/TiO_2_ Nano-composite was shown in (Fig. 2f). The corresponding optical diffraction pattern for Chitosan/TiO_2_ Nano-composite obtained by the selected area electron diffraction (SAED) is illustrated in (Fig. 2g). The lattice fringes are similar to that of the prepared TiO_2_, in addition to the presence of grains with smaller lattice fringes having an interlayer distance equal to 1.2 Å. This noted decrease in the interlayer spacing for the Chitosan/TiO_2_ Nano-composite may be due to confinement of chitosan within the TiO_2_ lattice. These results confirm the preparation of hetero-structure of Nano-composite material containing tri-phasic TiO_2_ immobilized effectively in biopolymer.

#### UV–visible DRS spectroscopy (optical properties)

**Fig. 3:**
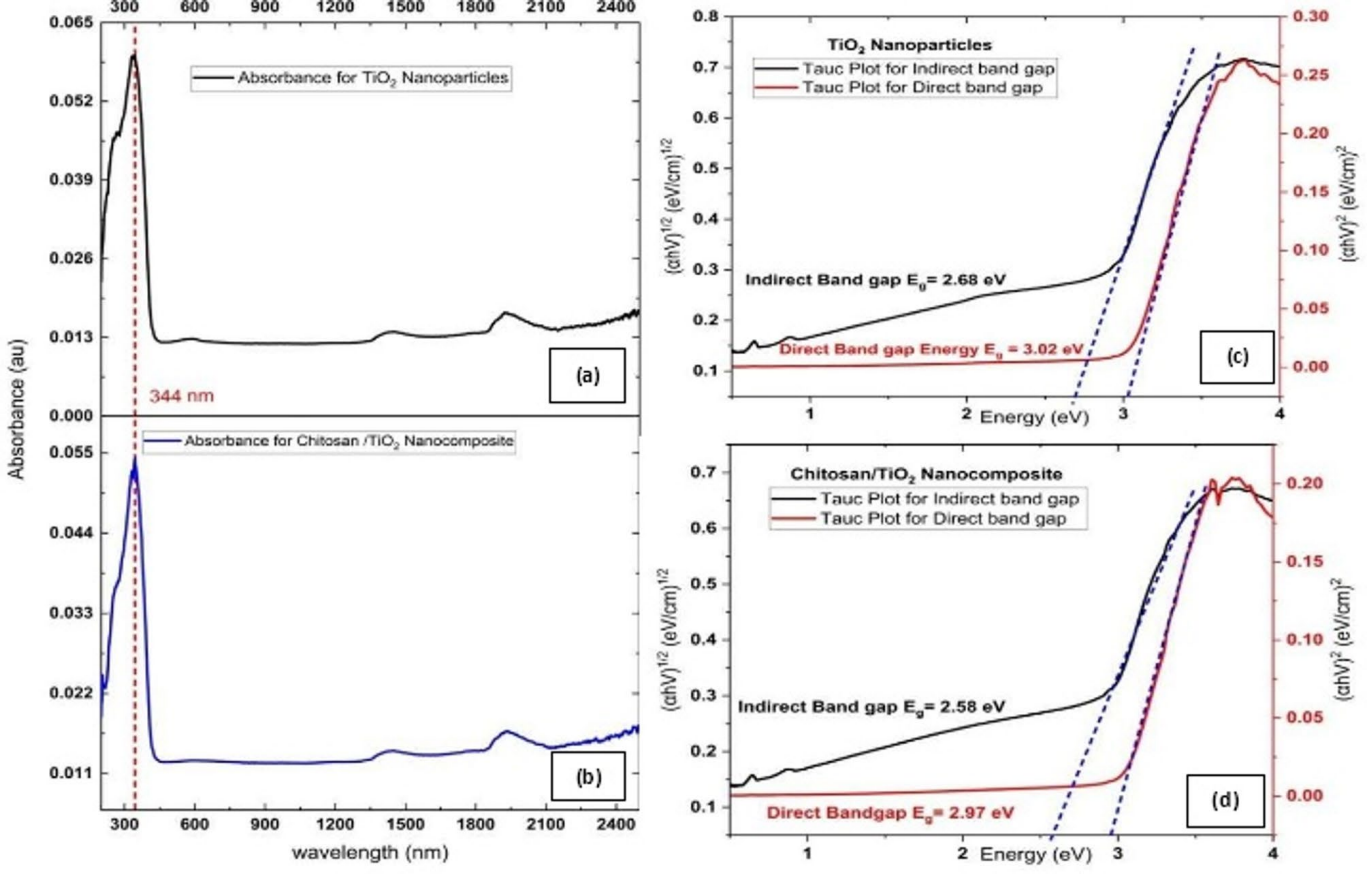
UV-Visible absorption spectra of (**a**) TiO_2_ Nano-particles, (**b**) chitosan/TiO_2_ Nano-composite; and band gap investigations analysis for (**c**) direct and indirect band gap plots for TiO_2_ Nano-particles, and (**d**) direct and indirect band gap plots for chitosan/TiO_2_ Nano-composite.

The investigations of the optical absorbance spectra of tri-phasic TiO_2_ Nano-particles and hetero-structure Chitosan/TiO_2_ Nano-composite were performed as illustrated in (Fig. 3a,b), respectively. The maximum absorption peak λ_max_ of TiO_2_ Nano-particles is 344 nm while Chitosan/TiO_2_ Nano-composite exhibits the same maximum absorption peak λ_max_ at 344 nm. A shoulder is noted at 0.06 au, and 0.054 au for tri-phasic TiO_2_ Nano-particles are noted at higher frequencies, and smaller peaks are noted at lower frequencies.

The band gap investigations for tri-phasic TiO_2_ Nano-particles and the immobilized TiO_2_ in chitosan biopolymer were illustrated in (Fig. 3c,d), respectively. The indirect band gap of tri-phasic TiO_2_ Nano-particles found to be 2.72 eV, while the indirect band gap for hetero-structure Nano-composite reduced to 2.58 eV. Furthermore, the direct band gap illustrated in (Fig. 3c,d) was slightly reduced for Chitosan/TiO_2_ Nano-composite and was found to be 2.97 eV, while for TiO_2_ Nano-particles found to be 3.02 eV. This slight reduction in band gap value implies that the Chitosan/TiO_2_ Nano-composite has slightly improved performance in sunlight absorption compared to the TiO_2_. It should be noted that the band gaps for pure anatase, rutile, and brookite phases are in the ranges 3.2–3.23, 3.02–3.04, and 3.1–3.3 eV, respectively^60,97^. The reduction of the value of the band gap of the prepared TiO_2_ is attributed to the anatase/rutile/brookite heterojunction that caused the red shift in the band gap of the prepared material^60^.

The calculated values are consistent with reported data on the polymorph TiO_2_^97–99^. The following Table 1 showed a comparison for the band gap values of various studied materials^100–105^. In particular, the doping of tri-phasic TiO_2_ Nano-particles with chitosan was reported to reduce the band gap from 3.2 eV to 3.1 eV; the value was further decreased by doping with Ce and chitosan to 2.8 eV. This value was attributed to the doping of the TiO_2_ lattice Ce, Ce and quantum dots. Hence, the further reduction of the band gap of the Nano-composites can be attributed to the doping of the TiO_2_ lattice with quantum dots. Such band gap narrowing is indicative of improved charge redistribution at the heterojunction interfaces, which can indirectly enhance electrostatic interactions with positively charged metal ions during sorption.

**Table 1:** Band gap values of various studied materials.

Material	Band gap	References
Chitosan/TiO_2_ nano-composite	Direct band gap 2.97 eV and indirect band gap 2.58 eV	This study
Fe_3_O_4_/CuO/CS nano-composite	Direct band gap 3.02 eV	^100^
CS: Mn metal complex	Direct band gap 1.77 eV and Indirect band gap 1.47 eV	^101^
CS–GO polymer nano-composites	3.8 eV	^102^
M^3+/^NaTiO_3_/PVA–Chitosan nano-composites (M = Ga, Ce, Nd or Er)	3.5 eV	^103^
Cs–MoS_2_ nano-composite	3.44 eV	^104^
Cs-TiO_2_, CS-Ag and CS-TiO_2_-Ag nano-composites	4.18, 2.82 and 2.74 eV	^105^

#### Effect of initial pH of solution on performance measures for Pb^2+^ and Cd^2+^

The effect of initial solution pH on the removal of Pb^2+^ and Cd^2+^ heavy metal contaminants onto the immobilized TiO_2_ in chitosan biopolymer material was studied at pH range of 3–11. In the removal process, the initial pH of solution is crucial as it might have an impact on both the protonation degree, the surface charge of the sorbent and the ionization process because it is affecting the solubility of heavy metal ions in solution^106,107^. Therefore, the importance of the investigation the influence of pH on removal of heavy metals ions like of Pb^2+^ and Cd^2+^ because of its impact on hydronium ion (H_3_O^+^) and speciation of Pb^2+^ and Cd^2+^ions to describe the availability of free Pb and Cd ions^62^. El Nagar and et al.^62^ discussed the impact of pH on the distribution of different heavy metals cations of aqueous chloride solution. They studied the hydrolysis of different heavy metals in the range of pH varying from (2–12) at constant temperature 298 K and constant ionic strength of 0.1 M. They found the predominance of free Pb^2+^ and Cd^2+^ at pH values 5.5 and 8, respectively. And when the pH values increased the cation hydroxide of each respective heavy metal increased on the expense of its corresponding free ion.

**Fig. 4:**
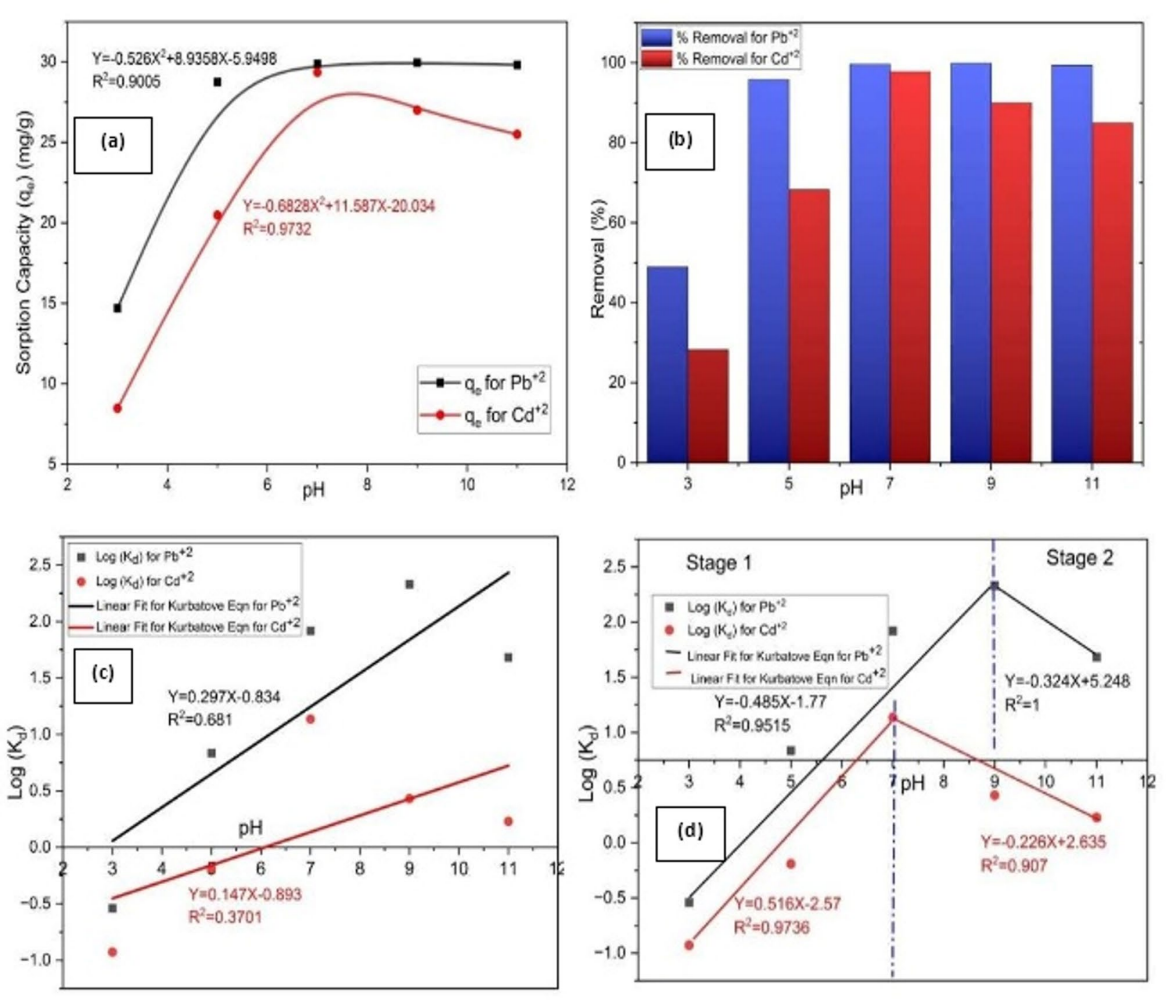
Influence for pH effect on performance measures for removal of Pb^2+^ and Cd^2+^ onto Chitosan/TiO_2_ nano-composite in terms of variance variables: (**a**) Sorption capacity (q_e_); (**b**) Removal efficiency (%); (**c**) Exchange reaction regression based on Kurbatov equation; and (**d**) Exchange reaction regression based on Kurbatov equation for two separate stages.

The results of effect of initial pH are presented in (Fig. 4); the (Fig. 4a) represented the effect of pH on removal capacity (q_e_) of Pb^2+^ and Cd^2+^. The data reveals that the removal efficiency of Pb^2+^ and Cd^2+^ onto Chitosan/TiO_2_ Nano-composite increased with increasing the pH values. While, (Fig. 4b) represented the effect of pH on removal efficiency percentage of Pb^2+^ and Cd^2+^.

For Pb^2+^ ions removal, the results indicate that in highly acidic solution, i.e. pH ≤ 5, rapid significant increase in the values of the removal capacity and efficiency are noted with increasing the pH. Where the removal efficiency increases sharply from 49 to 95.8% with the increase of pH from 3 to 5. At higher pH, both performance measures reach asymptotes. In this respect, neutral solution was chosen as the optimal for Pb^2+^ removals, where the removal capacity under this condition equals 29.892 mg/g.

For Cd^2+^ ions removal, the results exhibited significant increase in the removal efficiency from 28.25 to 97.85% by increasing the pH from 3 to 7 respectively. While, at pH > 7, the removal efficiency of Cd^2+^ onto chitosan/TiO_2_ Nano-composite decreased from 97.85% to 85%. Therefore, the highest removal efficiency observed at PH = 7 with optimum removal capacity equals 29.355 mg/g. From above mentioned results, it was indicated that the removal efficiency of Pb^2+^ was higher than the removal efficiency of Cd^2+^ within difference of 1.79% for the maximum removal percentage at pH 7.

The variation in removal behavior with changes in the solution acidity or alkalinity can be explained based on the surface charges characteristics of the chitosan/TiO_2_ Nano-composite as following:

- In highly acidic media, the high concentration of H^+^ ions compete with Pb^2+^ and Cd^2+^ ions for the available active sites on the composite surface, leading to reduce metal ion removal efficiencies^107^. Additionally, at low pH values, the functional groups present on the chitosan/TiO_2_ Nano-composite surface, undergo protonation, resulting in the formation of positively charged surface sites. This protonation reduces the electrostatic attraction toward metal cations. The protonation of surface can be described as follows Eq. (19)^108^.

19 $${\rm{Sur}} - {\rm{OH }} + {\rm{ }}{{\rm{H}}^ + } \to {\rm{ Sur}} - {\rm{O}}{{\rm{H}}_{\rm{2}}}^ +$$

- As the pH increase, the concentration of H^+^ ions decreased, thereby reducing their competitive effect and allowing greater availability of active sites for Pb^2+^ and Cd^2+^ removal. Under neutral and mildly alkaline conditions, deprotonation of the surface functional group occurs, generating negatively charged sites that favor the electrostatic attraction and surface complexation with of metal cations, leading to enhance removal efficiencies^109,110^. The deprotonation process can be represented as Eq. (20):

20 $${\rm{Sur}} - {\rm{OH }} + {\rm{ O}}{{\rm{H}}^ - } \to {\rm{ Sur}} - {{\rm{O}}^ - } + {\rm{ }}{{\rm{H}}_{\rm{2}}}{\rm{O}}$$

**Fig. 5:**
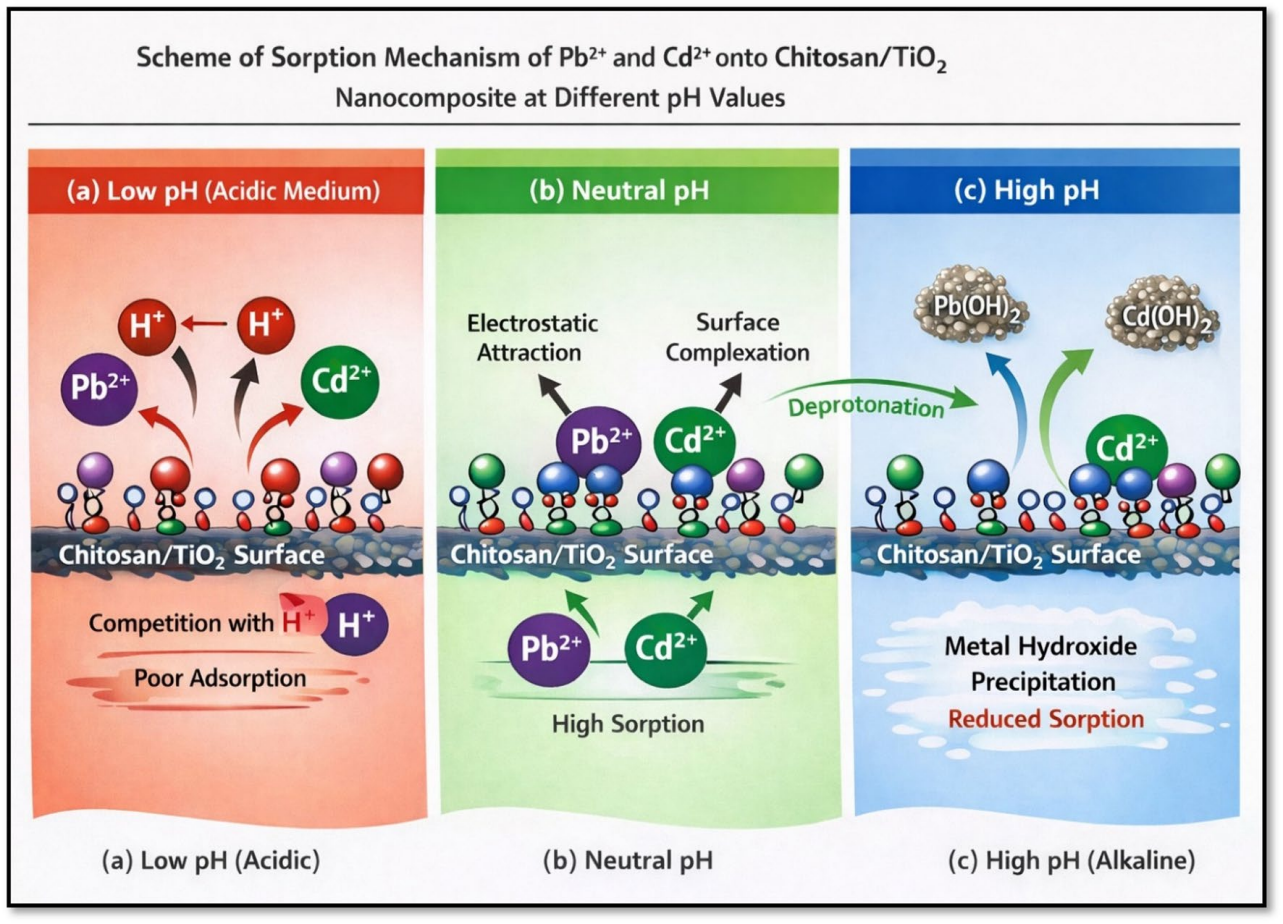
Schematic illustration of the proposed sorption mechanism of Pb^2+^ and Cd^2+^ ions onto chitosan/TiO_2_ Nano-composite at different pH values: (**a**) at low pH; (**b**) at neutral pH; and (**c**) at higher pH values.

However, at higher pH levels, metal ions may undergo partial precipitation in the form of metal hydroxides, such as of Pb(OH)_2_ for lead ions and Cd(OH)_2_, which contributes to an apparent decrease in sorption capacity [108]. Additionally, due to the presence of the fixed amount of metal chlorides, (MCl^-1^), which exist even at low pH, in addition to the buildup and decline of the hydroxyl phases[62]. The proposed sorption mechanism under different pH conditions is schematically illustrated in Fig. 5.

The equations which describe the effect of varying pH on removal of Pb^2+^ and Cd^2+^ were found to fit the following Eqs. (21), (22), (23) and (24):

- For Pb^2+^ ions:
  21 $${\rm{Removal capacity }}\left( {{{\rm{q}}_{\rm{e}}}} \right){\rm{ }} = {\rm{ }} - {\rm{ }}0.{\rm{526 p}}{{\rm{H}}^{\rm{2}}} + {\rm{ 8}}.{\rm{9358 PH }}{-}{\rm{ 5}}.{\rm{9498}},{\rm{ }}{{\rm{R}}^{\rm{2}}} = {\rm{ }}0.{\rm{9}}00{\rm{5}}$$
  22 $${\rm{Removal Efficiency }}\% {\rm{ }} = {\rm{ }} - {\rm{ 7}}.0{\rm{129 p}}{{\rm{H}}^{\rm{2}}} + {\rm{ 31}}.{\rm{595 pH }} + {\rm{ 71}}.0{\rm{92}},{\rm{ }}{{\rm{R}}^{\rm{2}}} = {\rm{ }}0.{\rm{9}}00{\rm{5}}$$

- For Cd^2+^ ions:
  23 $${\rm{Removal capacity }}\left( {{{\rm{q}}_{\rm{e}}}} \right){\rm{ }} = {\rm{ }} - {\rm{ }}0.{\rm{6828 p}}{{\rm{H}}^{\rm{2}}} + {\rm{ 11}}.{\rm{587 PH }}{-}{\rm{ 2}}0.0{\rm{34}},{\rm{ }}{{\rm{R}}^{\rm{2}}} = {\rm{ }}0.{\rm{9732}}$$
  24 $${\rm{Removal Efficiency }}\% {\rm{ }} = {\rm{ }} - {\rm{ 9}}.{\rm{1}}0{\rm{36 p}}{{\rm{H}}^{\rm{2}}} + {\rm{ 41}}.0{\rm{96 pH }} + {\rm{ 5}}0.{\rm{72}},{\rm{ }}{{\rm{R}}^{\rm{2}}} = {\rm{ }}0.{\rm{9732}}$$

The direct sensitivities of the removal capacity of both metal ions as a function of pH are given as follow Eqs. (25) and (26):

25 $$\frac{\partial{q}_{Pb}}{\partial pH}=-1.05pH+8.93$$

26 $$\frac{\partial{q}_{Cd}}{\partial pH}=-1.37pH+11.59$$

Hence, the direct sensitivity for the Nano-composite sorption capacity towards Pb will have maximum value at pH 3 equals 5.78 and minimum value at pH 11 equals − 2.62; indicating the direct sensitivity equal zero at pH equals 8.5. Similar behavior is noted for the direct sensitivity of the material toward the sorption of Cd with zero sensitivity at pH 8.5.

Theses experimental trends are well captured by Kurbatov equation. The plots of log K_d_ versus pH for Pb^2+^ and Cd^2+^ ions were represented in (Fig. 4c), while (Fig. 4d) represented the exchange reaction regression based on Kurbatov equation for two separated stages. The initial increase in sorption with rising pH, followed by plateau or decrease at higher pH, consistent with the observed performance measures. The calculated K_ex_ and n values are reported in Table 2 and the n values for Pb^2+^ ions are larger than that of Cd^2+^ ions indicating stronger interaction of Pb^2+^ with the Nano-composite surface compared to Cd^2+^, aligning with slightly higher removal efficiency of Pb^2+^. The n values lower than one, indicating that more than one mole of Pb or Cd was sorbed for the release of one mole of protons which is indicative of multiple reaction that occur during the removal process^111^. The calculated value of the correlation coefficient is very small due to the scatter of the experimental data in two distinctive trends, the first extend from highly acidic pH value to neutral value and the second extend from neutral value to highly alkaline value. The data shows that the logarithmic of the distribution coefficient of Pb^2+^ and Cd^2+^ onto Chitosan/TiO_2_ Nano-composite increased with increasing the pH values till reaching pH 9 and pH 7 for Pb^2+^ and Cd^2+^, respectively then decreased with increasing of pH values.

**Table 2:** Kurbatov stoichiometric parameters of H^+^/M^2+^ exchange for the removal of Pb^2+^ and Cd^2+^ ions onto Chitosan/TiO_2_ nano-composite.

Heavy metal Ions	Parameters	Values	
		Stage 1	Stage 2
Pb^2+^	n	0.485	− 0.324
	K_ex_	0.17	190.18
	R^2^	0.974	1
Cd^2+^	n	0.516	− 0.226
	K_ex_	0.076	13.94
	R^2^	0.974	0.907

#### Effect of contact time on performance measures for Pb^2+^ and Cd^2+^

**Fig. 6:**
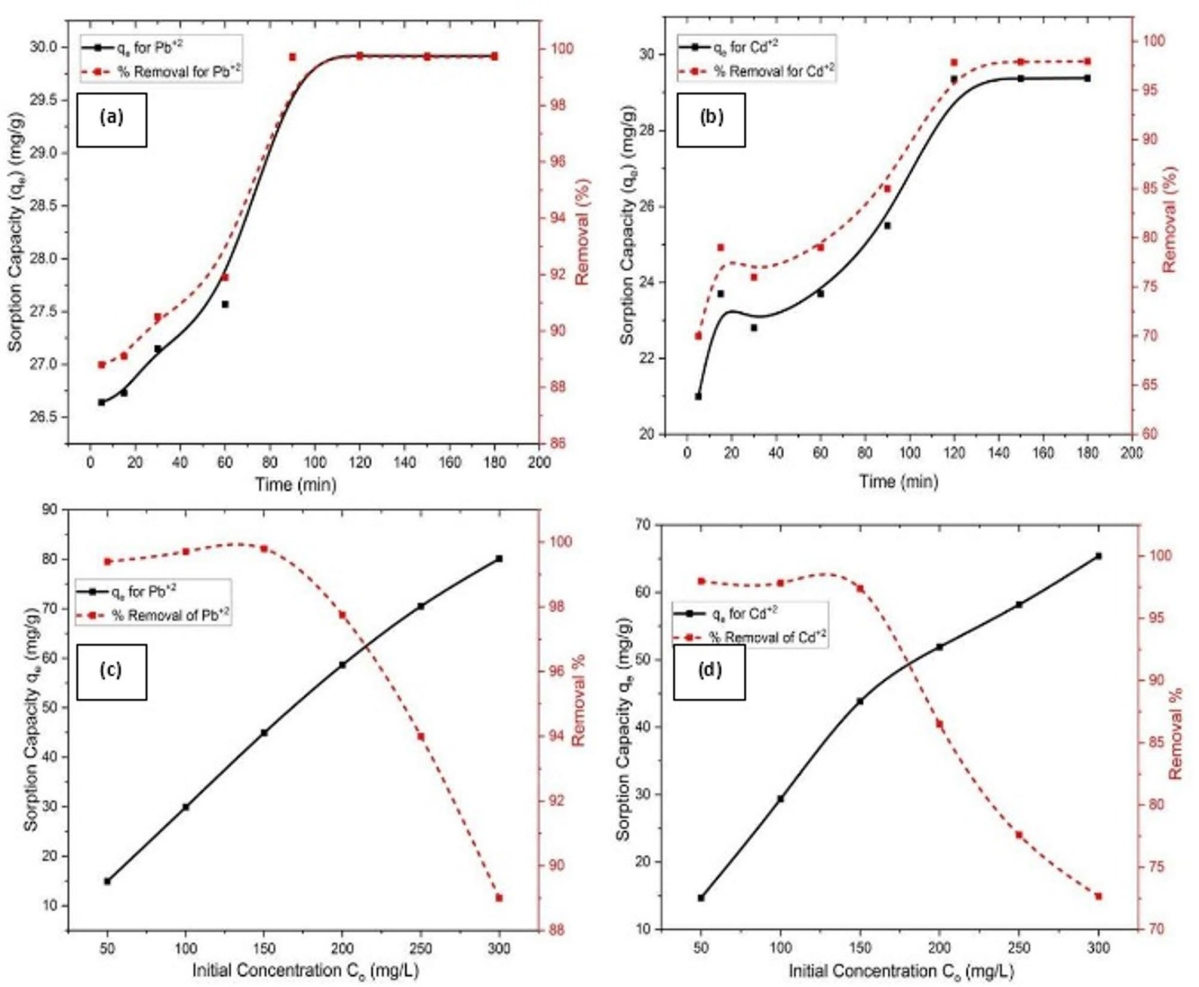
Influence for variance effects on performance measures for removal of Pb^2+^ and Cd^2+^ ions onto chitosan/TiO_2_ Nano-composite in terms of: (**a**) Contact time effect on Pb^2+^ removal; (**b**) Contact time effect on Cd^2+^ removal; (**c**) Initial concentration effect on Pb^2+^ removal; and (**d**) Initial concentration effect on Cd^2+^ removal.

The removal behavior of Pb^2+^ and Cd^2+^ by the immobilized TiO_2_ in chitosan biopolymer material as a function of contact time was illustrated in Fig. 6a,b, respectively. The hetero-structured Chitosan/TiO_2_ Nano-composite exhibited rapid metal uptake during the initial stages, followed by a gradual approach to equilibrium after 90 min for Pb^2+^ and 120 min for Cd^2+^, which can be attributed to the saturation of available sorption sites^65^.

During the first 5 min, Pb^2+^ removal was remarkably fast, achieving more than 88% removal efficiency with a removal capacity of 26.64 mg/g, whereas Cd^2+^ removal reached approximately 70%, corresponding to a removal capacity of 21 mg/g. Subsequently, a slower uptake stage was observed. The maximum removal capacity for Pb^2+^ reached 29.913 mg/g with a removal efficiency of 99.71% at 90 min, indicating that this time represents the equilibrium contact time and was therefore selected for subsequent Pb^2+^ removal experiments.

Similarly, Cd^2+^ removal reached equilibrium after 120 min, achieving a maximum removal capacity of 29.355 mg/g and a removal efficiency of 97.85%, which was adopted as the equilibrium contact time for further Cd^2+^ studies.

The removal kinetics of both Pb^2+^ and Cd^2+^ ions followed a two-step mechanism: an initial rapid uptake associated with external surface sorption through physical sorption^65^, followed by a slower stage governed by intraparticle diffusion of metal ions into the pores of Chitosan/TiO_2_ Nano-composite. This behavior is attributed to the high availability of active sites at the beginning of the process, which gradually decreases as these sites become occupied^62^. Consequently, the sorption rate diminishes due to increased competition among metal ions for the remaining active sites.

A comparative evaluation of the removal performance of various Nano-composite materials with the prepared Chitosan/TiO_2_ nano-composite for Pb^2+^ and Cd^2+^ removal is resented in Table 3^62,112–116^. Although some reported sorbents exhibit higher maximum sorption capacities, they often require longer contact times, harsh operating conditions, or lack sustainability aspects. In contrast, the prepared biopolymer-supported tri-phasic TiO_2_ nanocomposite demonstrates rapid equilibrium, high removal efficiency under sunlight, and ecofriendly characteristics, making it more suitable for practical water treatment applications.

**Table 3:** Comparison of Pb^2+^ and Cd^2+^ removal performance by different sorbent materials.

Heavy metal	Sorbent material	pH	Temp (°C)	V/m (L/g)	C_o_ (mg/L)	Contact time (h)	Q (mg/g)	References
Pb^2+^	Chitosan/TiO_2_ nano-composite	5	25	0.3	100	1.5	29.913	This study
	Nano-MFA-GP	5.5	60	0.05	1000	2.0	49.9	^62^
	Geo-FA/MK	3.0	25	0.25	300	10	73.5	^112^
	Geo-BFA	6.5	25	0.40	400	24	143.7	^113^
	MnFe_2_O_4_@SiO_2_@VTMS nano-composites	6	25	0.04	150	2	138.5	^114^
	MgO nanorods	3	25	2.5	-	24	116	^115^
Cd^2+^	Chitosan/TiO_2_ nano-composite	7	25	0.3	100	2	29.355	This Study
	Nano-MFA-GP	5.5	60	0.05	1000	2	47.5	^62^
	Geo-FA/MK	5.0	25	0.25	300	10	64.2–75.7	^112^
	Geo-S/dithiocarbamate	5.0	25	1.25	10	6	12.34	^116^
	Geo-BFA	6.5	25	0.40	80	24	26.84	^113^
	MnFe_2_O_4_@SiO_2_@VTMS nano-composites	6	25	0.04	150	2	130	^114^

#### Effect of initial concentration on performance measures for Pb^2+^ and Cd^2+^

The influence of an initial concentration for Pb^2+^ and Cd^2+^ ranging from 50 to 300 mg/L on the removal process, which is depicted in Fig. 6c,d. The figures show that when the initial concentration increased, the percentage of removal dropped due to the fixed amounts of sorbents utilized in the study and also due to the amount of metal ions surpassing the fixed number of available active sites^117^. That is because the initial concentration of metal ions offers sufficient driving force to overcome the resistance to metal ions mass transfer between the aqueous and solid phases. Additionally, increasing initial concentration improves the interaction with hetero-structure Chitosan/TiO_2_ Nano-composite. Consequently, the removal uptake of Pb^2+^ and Cd^2+^ is enhanced as the initial concentration of metal ions increases. This can be attributed to the increase in the concentration gradient’s driving force as the initial concentration rises.

Therefore, the percentage removal of Pb^2+^ was 89% at 300 mg/L of initial concentration, compared to 99.4% at 50 mg/L. While the percentage removal of Cd^2+^ decreased from 98% to 77.6% with an increase in initial concentration from 50 mg/L to 300 mg/L, respectively, as illustrated in Fig. 6c,d. In contrast, the removal capacity of heavy metal ions by Chitosan/TiO_2_ Nano-composite increases with the increase of initial concentration. This is due to the fact that the possibility of collisions with sorption sites on the sorbent’s surface increases with the initial concentration of heavy metal ions. Furthermore, the mass transfer driving force is improved, which helps to lower the resistance to mass transfer and raise the removal capacity^118^.

#### Kinetic study

**Fig. 7:**
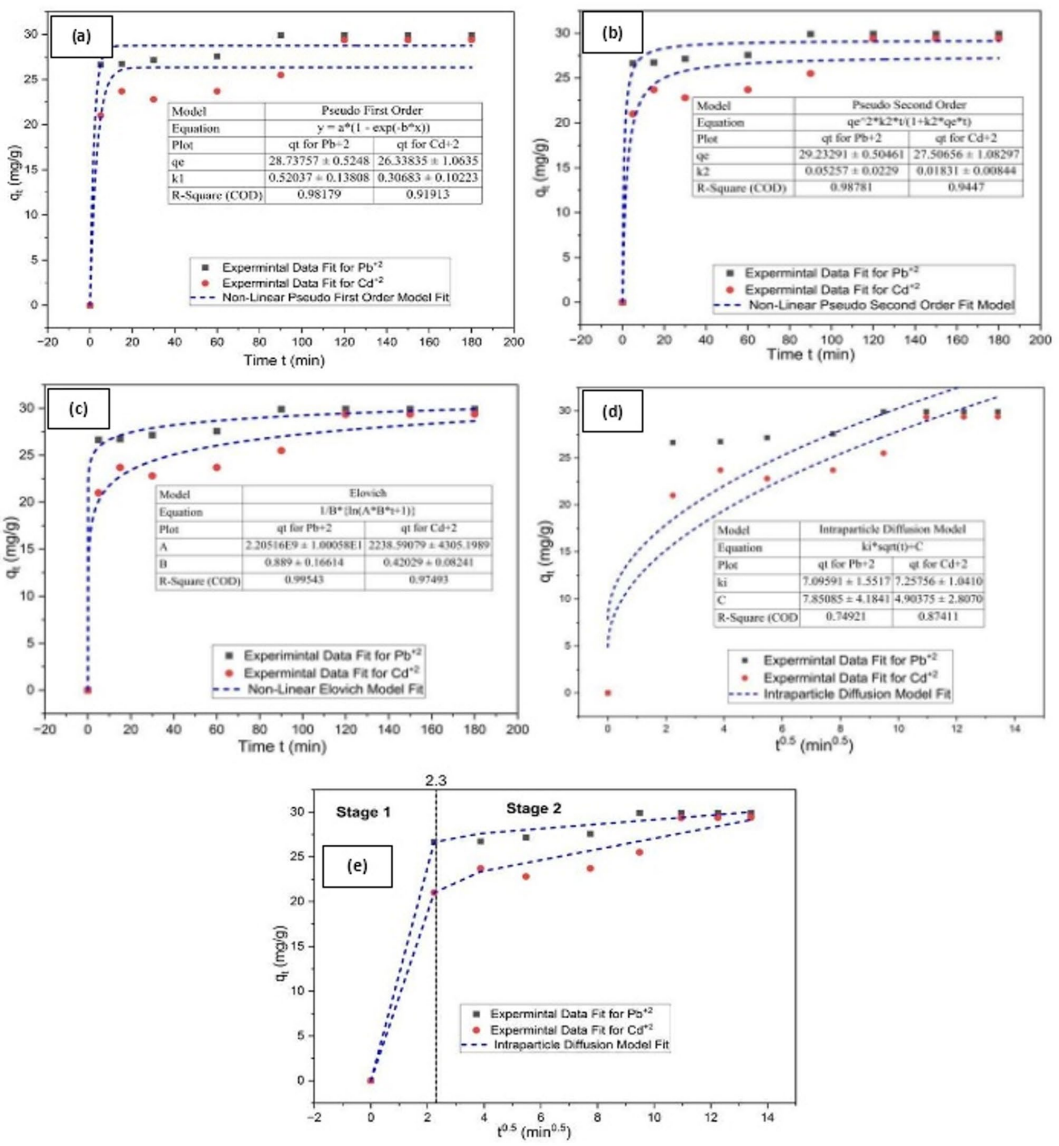
Kinetic study of Pb^2+^ and Cd^2+^ removal onto chitosan/TiO_2_ Nano-composite using different models (**a**) Pseudo-first order; (**b**) Pseudo-second order; (**c**) Elovich; (d) Intraparticle diffusion; and (**e**) Intraparticle diffusion for two separate stages.

The Pseudo-First order, Pseudo-Second order, Elovich, and Intraparticle diffusion kinetic models are studied and illustrated in Fig. 7a–d, respectively. And according to the correlation coefficients (R^2^) and the kinetic parameter listed in Table 4, the experimental data was well-fitted by the Elovich Model; the correlation value is closer to 1.0. This indicates that the removal process involved many mechanisms.

The intraparticle diffusion model was used to study the removal process in order to confirm the rate-controlling step. The removal process of Pb^2+^ and Cd^2+^ is depicted in (Fig. 7e) as a multiple mechanism including the sorption on the external surface and diffusion into the interior pores structure of the immobilized TiO_2_ in chitosan biopolymer material. The results indicate that the contaminants were removed onto Chitosan/TiO_2_ Nano-composite in two distinct stages, each of which would represent a controlling mechanism or many controlling mechanisms operating simultaneously^119^. The first quick stage (0 < t^1/2^ < 2.3), which extended during the first 15 min, was surface or film diffusion, which involves Pb^2+^ and Cd^2+^ ions in solution migrating to the surface of the Chitosan/TiO_2_ Nano-composite. That was due to the high availability of free sites on Chitosan/TiO_2_ Nano-composite surface. During this stage, considerable amounts of Pb^2+^ and Cd^2+^ were rapidly removed onto the surface of the Chitosan/TiO_2_ Nano-composite; this was mostly due to physical adsorption. After that at (t^1/2^ > 2.3) the availability of sites decreased and the removal process became more slower than before until reaching equilibrium, because a significant amount of sorbed Pb^2+^ and Cd^2+^ on the Chitosan/TiO_2_ Nano-composite needs to be further diffused into the hetero-structure Chitosan/TiO_2_ Nano-composite interior pores at the same time, contaminants are combined with the sorption active sites on the inner surface of the Chitosan/TiO_2_ Nano-composite, which are progressively removed by the Chitosan/TiO_2_ Nano-composite surface^120^. This process is responsible for the second region, which is a result of the intraparticle diffusion of Pb^2+^ and Cd^2+^ into the inner surface of the hetero-structure Chitosan/TiO_2_ Nano-composite. It involves both physical and chemical adsorption.

**Table 4:** kinetic parameters for Pb^2+^ and Cd^2+^ removal using chitosan/TiO_2_ nano-composite.

Kinetic model	Parameters	Values	
		Pb^2+^	Cd^2+^
Pseudo first order	K_1_ (min^− 1^)	0.520	0.307
	q_eq_ (mg/g)	28.738	26.338
	R^2^	0.982	0.919
Pseudo second order	K_2_ (g/mg.min)	0.053	0.018
	q_eq_ (mg/g)	29.233	27.507
	R^2^	0.988	0.945
Elovich	α (mg/g.min)	2.205 × 10^9^	2.239 × 10^3^
	β (g/mg)	0.889	0.420
	R^2^	0.995	0.975
Intraparticle diffusion	K_i1_ (mg/g.min^− 0.5^)	11.914	9.391
	K_i2_ (mg/g.min^− 0.5^)	0.250	0.605
	C (mg/g)	26.64	21
	R^2^	0.995	0.998

#### Sorption isotherms

**Fig. 8:**
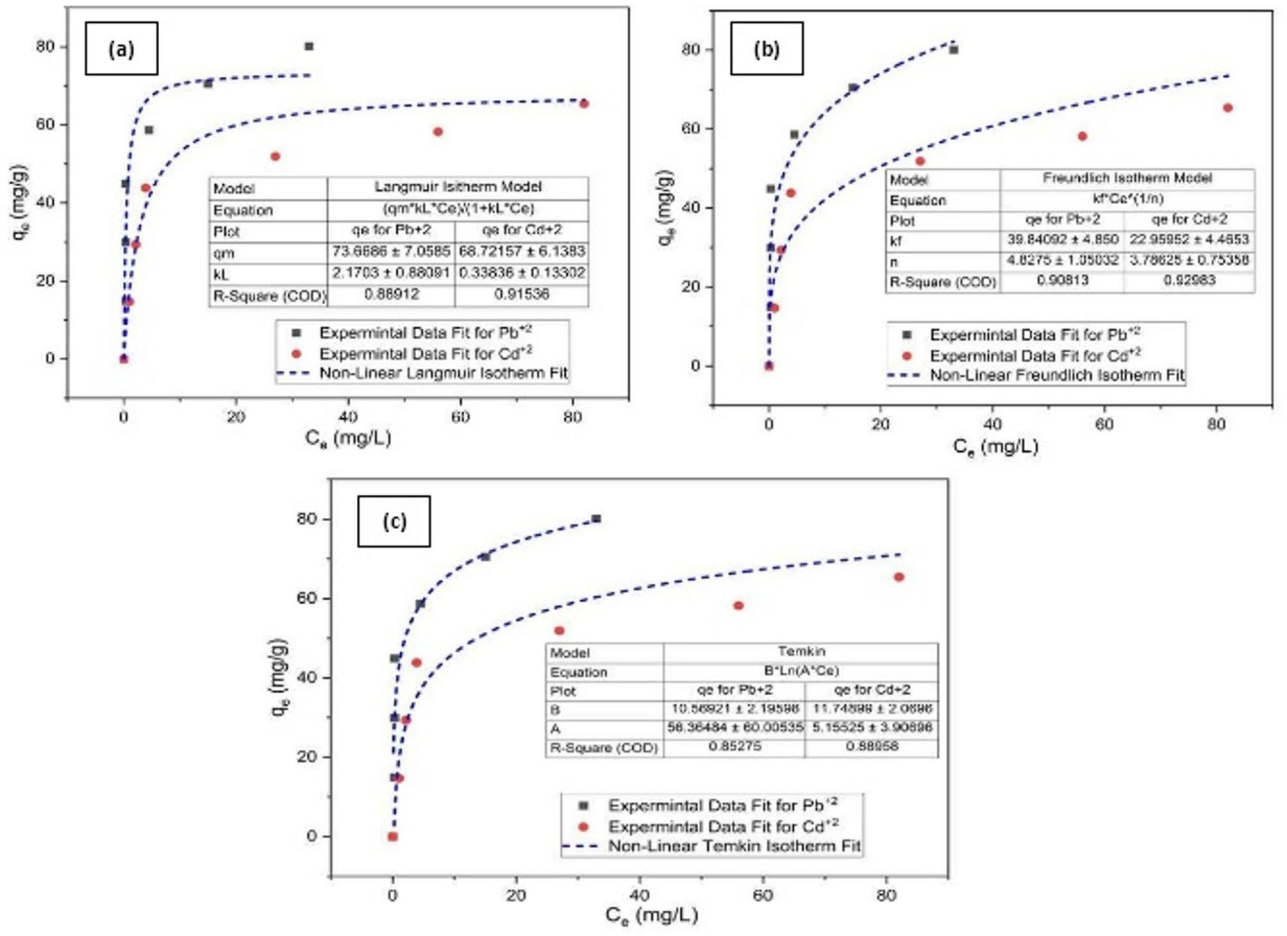
Sorption isotherms models for Pb^2+^ and Cd^2+^ removal onto chitosan/TiO_2_ Nano-composite: (**a**) Langmuir model; (**b**) Freundlich model; and (**c**) Temkin model.

The sorption isotherms for removal of heavy metal ion pollution (Pb and Cd) were tested at initial concentrations adjusted between 50 and 300 mg/L using a constant sorbent dosage of 0.1 g of the immobilized TiO_2_ in chitosan biopolymer material. The temperature of the solutions was maintained at 25 ℃ and pH 7 for both Pb^2+^ and Cd^2+^ ions solutions. The resulting data were transformed into non-linear fittings of Langmuir, Freundlich, and Temkin, which are displayed in Fig. 8a–c in that order. Table 5 displays several parameters that were determined from the sorption isotherms.

Since R^2^ is so close to 1, it is considered a measure of the fitting goodness of experimental data on the isotherm models^121^, which are nearly perfect in the Freundlich Model for sorption of Pb^2+^ and Cd^2+^ ions by Chitosan/TiO_2_ Nano-composite. Therefore, it can be deduced that multilayer adsorption with the interaction between adsorbed molecules is the mechanism by which the Pb^2+^ and Cd^2+^ ions are adsorbed onto Chitosan/TiO_2_ Nano-composite. This means that the metal ions are removed by heterogeneous energy sites that exist on the Chitosan/TiO_2_ Nano-composite surface in multiple layers^122,123^.

In optimum conditions, sorption capacities of Pb^2+^ and Cd^2+^ onto the hetero-structure Nano-composite were compared. The results showed that the maximum experimental removal capacity of different metal ions onto the Chitosan/TiO_2_ Nano-composite sorbent decreased in the order of Pb^2+^ (73.67 mg/g) > Cd^2+^ (68.72 mg/g) and was also compared to other sorbents from the literatures in Table 6^124-128^. The decrease in the maximum removal capacity may be related to the higher diffusion of Pb^2+^ ions onto the Chitosan/TiO_2_ Nano-composite and a similar investigation was reported by other researchers^129^.

In this study, the value of the adsorption intensity (R_L_) for Pb^2+^ and Cd^2+^ adsorption is in the range of (0–1), indicating that the process of adsorption of Pb^2+^ and Cd^2+^ heavy metal ions on the Chitosan/TiO_2_ Nano-composite is favorable. Therefore, the Chitosan/TiO_2_ Nano-composite can be used as a suitable sorbent for the uptake of Pb^2+^ and Cd^2+^ ions. This agrees with the findings regarding “n” values. For a number of studied systems, the range of advantageous adsorption in the Freundlich Model is represented by the n values, which vary from 1 to 10^130^. Metal ions were physically adsorbed onto the absorbent surface of Chitosan/TiO_2_ Nano-composite in this investigation, as shown by values of n ranging from 1 to 10, and this indicates that the Nano-composite is more favorable for the adsorption of Pb^2+^ and Cd^2+^ from their aqueous solutions^130^.

**Table 5:** Parameters of sorption isotherm models for Pb^2+^ and Cd^2+^ removal ions onto Chitosan/TiO_2_ Nano-composite.

Sorption isotherms models	Parameters	Values	
		Pb^2+^	Cd^2+^
Langmuir	K_L_ (L/mg)	2.17	0.34
	q_max_ (mg/g)	73.67	68.72
	R_L_	0.002	0.01
	R^2^	0.89	0.92
Freundlich	K_F_ (L/g)	39.84	22.96
	n	4.83	3.79
	R^2^	0.91	0.93
Temkin	B_T_	10.57	11.75
	A_T_ (L/g)	56.37	5.16
	B (J/mol)	234.42	210.88
	R^2^	0.85	0.89

**Table 6:** Comparison of maximum removal of Pb^2+^ and Cd^2+^ ions from contaminated water by using different sorbents.

Sorbents	Maximum removal capacity q_max_ (mg/g)		References
	Pb^2+^	Cd^2+^	
Chitosan/TiO_2_ Nano-composite	73.67	68.72	This study
Fe_3_O_4_/Graphene Oxide/Chitosan Nano-composite	63.45	–	^124^
Chemically Modified *Opuntia ficus indica* Cladodes	116.8	64.7	^125^
Theobroma cacao Agro-Industrial Waste	–	58.5	^126^
Eggshell/starch/Fe_3_O_4_ Nano-composite	57.14	48.53	^127^
Magnetic iron oxide chitosan (Fe_3_O_4_–CS) Nano-composite	23.75	12.38	^128^

#### Significance and outcomes of the present study

The significance of the present study lies in the development of an eco-friendly, multifunctional, and sunlight-active chitosan/TiO_2_ Nano-composite for efficient heavy metal detoxification from contaminated water. Unlike conventional systems that rely on single-phase TiO_2_ or require artificial UV irradiation, the current work demonstrates the successful immobilization of tri-phasic TiO_2_ (anatase, rutile, and brookite) within a chitosan matrix, forming a stable hetero-structure with enhanced interfacial interactions and improved performance under natural sunlight.

The coexistence of the three TiO_2_ polymorphs promotes effective charge separation and reduces electron–hole recombination, and exposes high-energy surface facets that enhance metal ion binding. Meanwhile, chitosan provides abundant amino and hydroxyl functional groups for metal binding. This synergistic combination leads to high sorption capacities, rapid attainment of equilibrium, and superior removal efficiencies for Pb^2+^ and Cd^2+^ ions under mild, sunlight-driven conditions.

Furthermore, the strong interfacial coupling between TiO_2_ nanoparticles and the chitosan matrix suggests good structural stability, indicating the potential recyclability of the developed nanocomposite. The preferential interaction of Pb^2+^ and Cd^2+^ with the functional groups of chitosan and surface-active TiO_2_ facets also implies favorable selectivity toward toxic heavy metals over competing ions.

Overall, the outcomes of this study demonstrate that the Chitosan/TiO_2_ Nano-composite is not only highly effective in heavy metals removal but also offers practical advantages, including cost-efficient, environmental sustainability, and reduced energy demand. These features make the developed material a promising candidate for real-world water treatment applications, particularly in resource-limited regions where sunlight-driven and environmentally benign technologies are highly desirable.

## Conclusion

In this study, a tri-phasic polymorph TiO_2_ Nano-material was successfully synthesized via a sol–gel method and chemically immobilized within a chitosan biopolymer matrix, yielding a stable hetero-structure Nano-composite for heavy metal detoxification. The material exhibited excellent performance for the elimination of Pb^2+^ and Cd^2+^ ions from their aqueous solutions under sunlight.

Unlike previously reported chitosan/TiO_2_ systems that mainly employ single- or biphasic TiO_2_, the present work demonstrates the effective integration of anatase, rutile, and brookite phases (A_34.6_R_56.8_B_8.6_), which enhances interfacial interactions and improves sunlight-responsive properties. UV-vis DRS analysis revealed a reduction in both direct and indirect band gap after immobilization within the chitosan matrix, indicating enhanced light absorption under sunlight.

The developed chitosan/TiO_2_ Nano-composite achieved high removal efficiencies of 99.86% for Pb^2+^ and 97.85% for Cd^2+^ ions at an optimal pH of 7, with equilibrium attained within 90 min for Pb^2+^ and 120 min for Cd^2+^. The sorption process followed the Elovich kinetic model, suggesting heterogeneous surface-controlled mechanisms. According to the Langmuir isotherm model, the maximum monolayer sorption capacities of 73.67 and 68.72 mg/g for the removal of Pb^2+^ and Cd^2+^ ions, respectively. Overall, this work advances the design of the biopolymer-supported Nano-composites by highlighting the role of tri-phasic TiO_2_ heterostructures in enhancing sorption performance and sunlight responsiveness, offering a sustainable and eco-friendly approach for heavy metal removal from contaminated water.

Although detailed recyclability and selectivity experiments were beyond the scope of the present study, the strong immobilization of TiO_2_ within the chitosan matrix and the nature of metal–functional group interactions suggest good structural stability, reusability potential, and selective affinity toward toxic divalent metal ions. Moreover, the developed Nano-composite is inherently energy-efficient due to sunlight-driven operation and economically favorable owing to the use of low-cost, naturally abundant materials.

From a future perspective, further investigations should focus on systematic recyclability assessment, selectivity studies in multi-ion systems, scalability evaluation, reusability and comprehensive techno-economic analysis under real water treatment conditions. Furthermore, future work could include a quantitative assessment of energy consumption and a full economic evaluation to support large-scale applications. These efforts may contribute to the development of sustainable, low-cost, and sunlight-responsive materials for large-scale water purification applications.

## Data Availability

The datasets generated and/or analyzed during the current study are included in this article.
